# Treatment of Insulinomas by Laparoscopic Radiofrequency Ablation: Case Reports and Literature Review

**DOI:** 10.1515/med-2020-0013

**Published:** 2020-02-11

**Authors:** Changyu Yao, Xiangtao Wang, Yongli Zhang, Jian Kong, Jun Gao, Shan Ke, Xuemei Ding, Zonghai Xin, Wenlei Xu, Shaohong Wang, Wenbing Sun

**Affiliations:** 1 Department of Hepatobiliary Surgery, Beijing Chaoyang Hospital Affiliated to Capital Medical University, No. 5, Jingyuan Street, Beijing 100043, China; 2 Department of General Surgery, Zhanhua People’s Hospital, Shandong Province, China

**Keywords:** Insulinoma, Radiofrequency ablation, Laparoscopy, Safety, Efficacy

## Abstract

Despite its rarity, insulinoma is the most common type of pancreatic endocrine neoplasm, with an occurrence of 1 to 5 per million per year in the population. Surgical resection or enucleation is the first line of curative treatment choice for insulinoma. Eight patients with symptomatic insulinomas treated by radiofrequency ablation have been described since 2009. In the past two years, we treated two patients with symptomatic insulinomas (one in the pancreatic tail and the other in the pancreatic neck) successfully using laparoscopic radiofrequency ablation. Both patients achieved complete elimination without any significant complications. Our study suggests laparoscopic radiofrequency ablation could be developed as a safe and effective alternative treatment to surgery for the patients with insulinomas who refuse or are not eligible for surgery.

## 1 Introduction

Despite its rarity, insulinoma is the most common type of pancreatic endocrine neoplasm, with an occurrence of 1 to 5 per million per year in the population [1]. More than 90% of insulinomas are sporadic, usually small (<20 mm) and benign (>90%) and occur throughout the pancreas. The diagnosis of insulinoma is often delayed as there may be a myriad of symptoms associated with neuroglycopenia, including confusion, neuropathies, blurred vision and an adrenergic response. Insulinoma may occur throughout life and afflict both genders equally. Anatomical distribution may vary throughout any part of the pancreas, but they are predominantly located in the pancreatic body and tail [2]. Surgical resection or enucleation is the first line of curative treatment choice for insulinoma [3,4]. The adoption of surgical procedures required are dependent on the anatomical position and size of the tumor. The operation may vary from a distal pancreatectomy, to enucleation of the tumor, or to a pancreaticoduodenectomy. However, a postoperative morbidity occurred in 35.4% or 32.8% of patients for open or laparoscopic surgical management respectively [5]. The medical treatment, such as administration of diazoxide or trans-catheter arterial embolization (TAE) are therapeutic options for patients who refuse or are not candidates for surgery. In the recent decade, radiofrequency ablation (RFA) was used as a method of multimodality therapy for liver and other solid tumors [6] and the first choice for some well-selected early-staged tumors. RFA may be an ideal curative alternative therapy to surgery for insulinomas, since they are usually benign, solitary and small at the time of diagnosis. In 2009, Limmer *et al*. [7] described the first case of insulinoma which was successfully treated by RFA, followed by reports of additional seven cases of patients with the same treatment [8, 9, 10, 11, 12]. In the past two years, we evaluated and documented the safety and efficacy of laparoscopic RFA for the treatment of two cases of patients with insulinomas

and reviewed the literatures associated with this novel treatment strategy (Table 1) .

## 2 Case one

**Figure 1:**
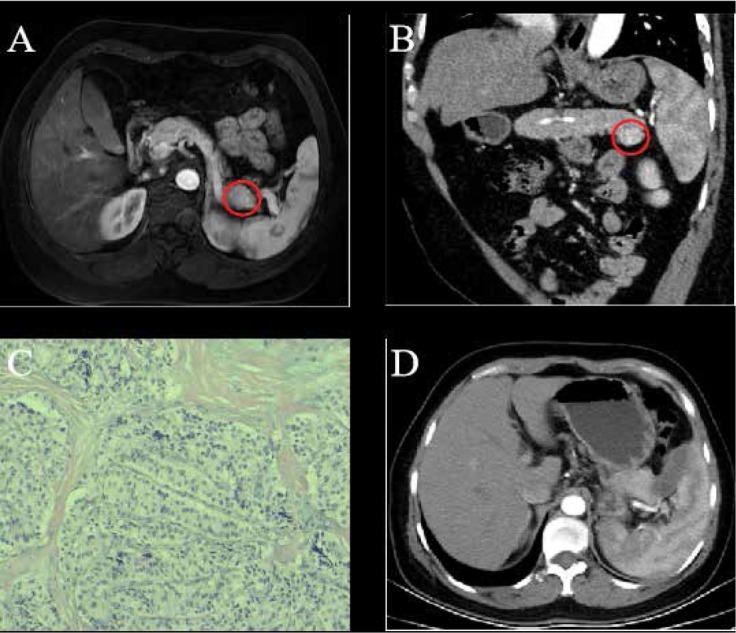
A 44-year-old female with pathology-proven pancreatic insulinoma was treated with laparoscopic RFA. (A) Contrast-enhanced MRI showed a round-like, well-defined lesion in the pancreatic tail in the arterial phase (red cycle). (B) Coronal reconstructed CT image showed the tumor grew in an exophytic pattern (red cycle). (C) Pathology confirmed the diagnosis of pancreatic insulinoma (HE×100). (D) Contrast-enhanced CT showed no residual tumor foci and recurrent lesion with a small volume of fluid collection near the pancreatic tail.

A 44-year-old female patient who had episodic hypoglycemic symptoms including sweating, palpitations and weakness for more than four years was referred to hospital. Physical examination didn’t show any abnormalities of heart, lung, kidney and other organs. Her random blood glucose was 2.4 mmol/L, fasting blood glucose was 1.7 mmol/L, glycosylated hemoglobin was 3.3% and fasting serum insulin was 372.6 mU/L. Contrast-enhanced magnetic resonance imaging (MRI) showed a round-like and well-defined lesion in the pancreatic tail with measured diameters of 18 mm × 17 mm in the arterial phase (Fig. 1 A). A coronal reconstructed CT scan showed that the tumor grew in an exophytic pattern (Fig. 1 B). The preoperative diagnosis of functioning insulinoma of the pancreatic tail was made based on the clinical symptoms, results of laboratory tests and characteristic MRI and CT imaging features.

Laparoscopic RFA was performed for the patient under general anesthesia. After the incision of the gastrocolic ligament made, an 18 mm tumor was visualized in the pancreatic tail corresponding to the tumor lesion depicted on CT and MR images. Before the RFA, a piece of tumor tissue was excised for the intraoperative pathologic examination, which confirmed the diagnosis of a benign lesion. Subsequently, a Cool-tip ACTC 2020 needle was placed in the lesion under the laparoscopic guidance with the tip of the needle being positioned in the center of the tumor. The ablation was carried with a Covidien Healthcare (Ireland, Dublin) generator with the power set at up to 40 W for the whole procedure. An abdominal drain was kept in the ablation region after the RFA procedure. The follow-up laboratory test showed the blood glucose was 7.6 - 9.8 mmol/L on the first day post the RFA procedure. No more hypoglycemia-related symptoms such as sweating, palpitation and weakness were seen. The level of fasting and random blood glucose was 5.3 mmol/L and 5.7 mmol/L respectively, the glycosylated hemoglobin was 4.3% and fasting serum insulin was 63.4 mU/L.

No pancreatic fistula occurred after the abdominal drain was withdrawn on the third postoperative day and the patient was discharged seven days later after the RFA procedure without any RFA-related complications. The pathological examination further confirmed the pre-procedural diagnosis of pancreatic insulinoma (Fig. 1 C). No recurrence of episodic hypoglycemic symptoms such as sweating, palpitation and weakness were observed in this patient during a 9-month follow-up period. Contrast-enhanced CT showed no residual tumor foci and a recurrent lesion with a small volume of fluid collection near the pancreatic tail. (Fig. 1 D).

## 3 Case two

**Figure 2:**
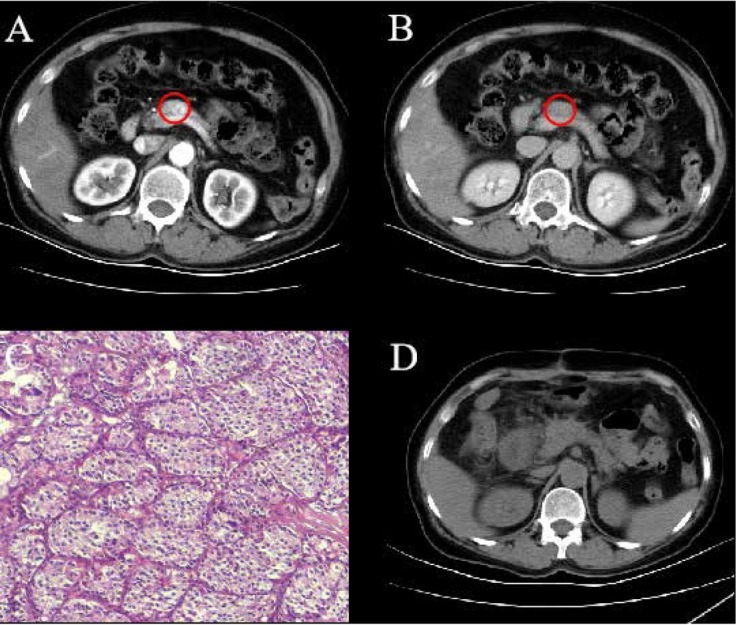
A 65-year-old female with insulinoma in the neck of the pancreas was treated with laparoscopic RFA. (A) Contrast-enhanced CT scan depicted a markedly enhanced lesion in the neck of the pancreas in the early arterial phase (red cycle). (B) Contrast-enhanced CT scan showed an isointense lesion in the delayed phase (red cycle). (C) Pathology confirmed the diagnosis of pancreatic insulinoma (HE×100). (D) The postoperative CT scan suggested the tumor was completely ablated.

A 65-year-old female complaining for more than 3 years of episodic hypoglycemic symptoms including recurrent sweating, palpitation and weakness was referred to hospital. No obvious abnormalities of her heart, lung, kidney and other major organs were found by physical examination. The results of routine laboratory test of blood, urine and stool routine were normal. The level of blood glucose was 2.3 mmol/L, carcinoembryonic antigen (CEA) was 6.5 μg/L, and carbohydrate antigen (CA) 19-9 was 67.5 kU/L. The contrast-enhanced CT scan depicted a markedly enhanced 15 mm lesion in the neck of the pancreas in the early arterial phase and an isointense lesion in the delayed phase (Fig. 2 A & B). A preoperative diagnosis of functioning insulinoma of the pancreatic neck was made based on clinical symptoms, laboratory results and the featured CT images.

A laparoscopic RFA was performed for the patient under general anesthesia. After a thorough exploration of the pancreas, a well-demarcated 15 mm tumor with complete capsule was found in the inferior aspect of the pancreatic neck. The pathologic examination of a piece of lesion tissue showed the histology of a benign lesion. Subsequently, RFA was performed for the tumor with the same ablation. After that, an abdominal drain was kept in the ablation region. The level of blood glucose rose to 6.1 - 8.2 mmol/L on the first day after the treatment. The patient no longer experienced the hypoglycemia-related symptoms, such as sweating, palpitation and weakness and the fasting and random blood glucose were normal.

No pancreatic fistula was observed after the abdominal drain was withdrawn on the third postoperative day, and the patient was discharged at the sixth day after the RFA procedure. The detailed pathology further confirmed the diagnosis of pancreatic insulinoma (Fig. 2 C). The patient didn’t experience episodes of hypoglycemia-related symptoms such as sweating, palpitation and weakness during a 43-month follow-up period. The postoperative CT scan suggested the tumor was completely ablated (Fig. 2 D) .

**Table 1:** Summary of RFA for insulinmoas of ten patients

References	Age (y)	Sex	Clinical presentation	Tumor location	Tumor size (mm)	Techniques of ablation	Postoperative complications	Follow-up time (m)	Recurrence	Result
Limmer [7]	80	F	episodes of severe hypoglycemia	tail	15	percutaneous puncture	no	7	no	complete ablation
Akhlaghpoor [8]	48	M	recurrent symptoms of dizziness, hunger, perspiration, and nervousness	head	12	percutaneous puncture	no	36	no	complete ablation
Procházka [9]	75	F	episodic hypoglycemic symptoms	body	15	laparoscopic guidance	transitory hyperglycemia	3	no	complete ablation
Waung[10]	70	F	recurrent episodes of dizziness for 18 m	uncinate process	18	EUS	no	10	no	complete ablation
Lakhtakia [11]	42	M	hypoglycemia with recurrent episodes of seizures for 4 y	body	14x12	EUS	no	12	no	complete ablation
	41	M	hypoglycemia with frequent eating and significant weight gain for 1 y	head	17x12	EUS	no	12	no	complete ablation
	52	M	hypoglycemia with recurrent episodes of syncope for 2 y	head, body, tail	22x19	EUS	no	11	no	complete ablation
Bas-Cutrina [12]	63	F	periodic hypoglycemic episodes	body	9x10	EUS	no	10	no	complete ablation
Sun	44	F	episodic hypoglycemic symptoms for 4 y	tail	18x17	laparoscopic guidance	no	9	no	complete ablation
Sun	65	F	episodic hypoglycemic symptoms for 3 y	neck	15x15	laparoscopic guidance	no	43	no	complete ablation

M: male, F: femal

Ethical approval: The research related to human use has been complied with all the relevant national regulations, institutional policies and in accordance the tenets of the Helsinki Declaration, and has been approved by the authors' institutional review board or equivalent committee.

Informed consent: Informed consent has been obtained from all individuals included in this study.

## 4 Discussion

Our study described the management of two patients with solitary and benign insulinomas by the treatment modality of laparoscopic RFA. No postoperative complications and tumor recurrence were observed during the follow-up period, which shows the feasibility, efficacy and safety of using laparoscopic RFA as alternative treatment to surgery for insulinoma.

Therapeutic strategies for insulinoma typically include surgical resection, medical treatment, TAE and ablative therapies. Surgical management is the mainstay of treatment modality for insulinoma and surgical resection or enucleation is the first choice of established curative therapy, but the postoperative morbidity rate of open or laparoscopic surgical management was 35.4% or 32.8% respectively, the mortality rate of open approach was 3.7% and the recurrence rate was 7.2% [5]. To perform the pancreatic resection safely and effectively requires considerable training, but the surgical treatment of insulinoma is only available in high profile medical centers. Medical treatment is usually prescribed for the patients who refuse the surgical resection or are not candidates for surgery. However, studies showed that drugs, such as diazoxide and octreotide, have various side effects and only a minority of patients can achieve the long-term improvement of hypoglycemia [13,14]. TAE is an effective procedure which is recommended for patients who are not eligible for or refuse surgery. Post-procedure complications of TAE including abdominal pain, mild pancreatitis and transitory mild diabetes were observed [15, 16, 17, 18, 19].

Local ablative therapies have been performed for patients with insulinoma who are not eligible for or refuse surgical management, including ethanol ablation (EA), microwave ablation (MWA), high intensity focused ultrasound ablation (HIFU), irreversible electroporation (IRE) and RFA. The postoperative complications of EA for insulinomas includes localized abdominal pain, mild pancreatitis, pseudocysts and duodenal ulceration, and disease recurrence was observed in four patients among twenty-six patients treated with EA [20, 21, 22, 23, 24, 25, 26, 27, 28, 29, 30]. EA can only be eligible for small lesions, and it has the contraindication for the tumor in the proximity to blood vessels and poses the risk of hemorrhage [25]. Chen *et al*. [31] described the treatment of one patient with insulinoma by CT-guided percutaneous MWA, demonstrating that MWA can yield a higher efficacy than RFA and is more suitable for bigger pancreatic lesions. Orgera *et al*. [32] described the treatment of two patients with insulinomas with HIFU and suggested this therapeutic modality should be prescribed for patients who are not candidates for surgery or other minimally invasive treatment. Papamichail *et al*. [33] reported one insulinoma patient treated with IRE developed mild pancreatitis postoperatively and suggested this novel non-direct thermal ablative technique was recommended for patients who are not eligible for surgical resection or other conventional ablative treatments [34].

**Figure 3:**
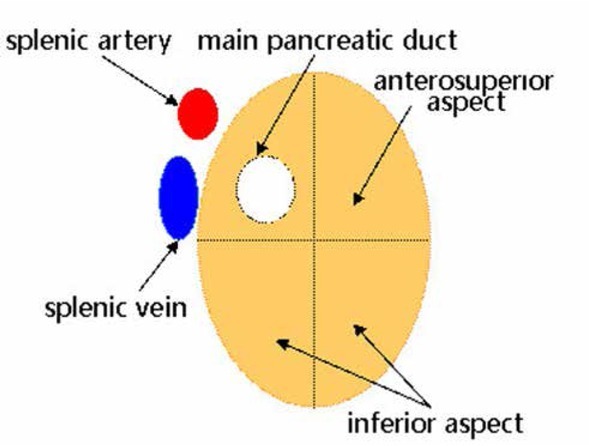
The safe region of pancreatic RFA is the anterosuperior and inferior aspects of the pancreas.

RFA is considered a safe and potentially curative technique for the treatment of hepatic tumors. More than 90% of insulinomas are benign, solitary, and small (<20 mm) at the time of diagnosis, and the main pancreatic duct is usually located in the posterosuperior region of the pancreas, so it is safe to perform RFA for insulinomas originating in the anterosuperior and inferior aspect of the pancreas (Figure 3). Limmer *et al*. [7] firstly reported one patient with insulinoma was successfully treated with CT-guided percutaneous RFA, with the result showing that percutaneous RFA can be performed safely for insulinoma by experienced doctors. However, the pancreas is a deeply located retroperitoneal organ and RFA performed in the pancreas poses the risk of accidental puncturing and thermal injuries of adjacent critical anatomic structures, such as the stomach, duodenum, transverse colon, common bile duct, portal vein, mesenteric and splenic vessels, which determines the difficulty of this procedure in pancreatic head [35]. RFA assisted by endoscopic ultrasound (EUS) for treating insulinoma enables the easier target of the tumor lesion than the percutaneous approach. Lesions located in pancreatic body or tail can be targeted through the stomach and lesions located in pancreatic head can be accessed by puncturing through the duodenum. In studies by Lakhtakia *et al*. [10, 11, 12], five patients with insulinomas were treated with RFA under the EUS-guidance. No major postoperative complications and disease recurrence were observed. However, EUS-guided RFA for insulinomas can damage the gastrointestinal tract and it is necessary to be performed by experienced endoscopic experts in high-profile medical centers. Procházka *et al*. [9] firstly described the successful treatment of one insulinoma with laparoscopic RFA assisted with intraoperative ultrasound. The long-term follow-up didn’t find any postoperative complications. In our current study, two patients with insulinomas were treated by laparoscopic RFA. Both of the patients achieved the complete elimination of the tumor and no postoperative complications occurred. The laparoscopic RFA has a few advantages: (i) direct visualization of the tumors and pancreas can be offered by laparoscopic view, which could minimize the damage of the adjacent structures [4,36]; (ii) laparoscopy is a minimally invasive technique but has a high diagnostic accuracy and tissues can sampled for cytopathology and immunohistochemistry, which is helpful for the differentiation between benign and malignant tumors and the adoption of appropriate management; (iii) an abdominal drain could be placed in the ablation region intraoperatively in case of the occurrence of pancreatic fistula. Although these two patients were treated safely and effectively by the laparoscopic RFA, the long-term benefits and the potential risks need to be investigated in a large series of patients with long follow-up periods.

In our practice, indications of RFA for insulinoma are strictly controlled. The location of the lesion is the crucial factor when we perform RFA as the first choice for insulinoma. Insulinoma located in the pancreatic tail is the best indication of RFA and RFA can also be performed for lesions located on the surface of pancreatic head, neck, and body with a rather safe distance to the main pancreatic duct. Lesions located deeply in the pancreas parenchyma and closed to the main pancreatic duct should be considered as contraindications of RFA and surgical treatment could be performed as the first choice for these lesions, including segmental resection, distal pancreatectomy (spleen preserving), and pylorus-preserving Whipple [5].

In conclusion, our clinical preliminary observation of two female patients with insulinomas treated with laparoscopic RFA suggests that this procedure is technically feasible, safe and effective for the patients with insulinomas who refuse or are not eligible for surgery. However, multicenter studies involving a larger series of patients with longer follow-up periods are essential to assess the safety and efficacy of this method as the curative alternative treatment to surgery for insulinoma.
